# Molecular docking analysis of UniProtKB nitrate reductase enzyme with known natural flavonoids

**DOI:** 10.6026/97320630012425

**Published:** 2016-12-27

**Authors:** Ayub Shaik, Vishnu Thumma, Aruna Kumari Kotha, Sandhya Kramadhati, Jalapathy Pochampally, Seshagiri Bandi

**Affiliations:** 1 Department of Chemistry, University College of Science, Osmania University, Hyderabad, Telangana, India; 2 Department of Sciences and Humanities, Matrusri Engineering College, Hyderabad, Telangana, India; 3 Bioinformatics Division, Osmania University, Hyderabad,Telangana, India

**Keywords:** Homology modeling, nitrate reductase, natural flavonoids, docking

## Abstract

The functional inference of UniProtKB nitrate reductase enzyme (UniProtKB - P0AF33) through structural modeling is of interest in plant biology. Therefore, a homology model for UniProtKB variant of the enzyme was constructed using available data with the MODELER software tool. The model was further docked with five natural flavonoid structures such as hesperetin, naringenin, leucocyanidin, quercetin and hesperetin triacetate using the AUTODOCK (version 4.2) software tool. The structure aided molecular interactions of these flavonoids with nitrate reductase is documented in this study. The binding features (binding energy (ΔG) value, H bonds and docking score) hesperetin to the enzyme model is relatively high, satisfactory and notable. This data provides valuable insights to the relative binding of several naturally occurring flavonoids to nitrate reductase enzyme and its relevance in plant biology.

## Background

Nitrogen is one of the most important growth-limiting nutrients in plants. The major source of nitrogen in most of the higher plants is nitrate (NO3) absorbed through roots. Nitrate can be reduced both in the photosynthetic tissues and in non-photosynthetic tissues such as roots [1]. Nitrate reductase catalyses the oxidation of NAD(P)H and the reduction of nitrate to nitrite [2]. This is subject to control at the levels of enzyme activity, synthesis, and degradation [3]. Nitrate reductase catalyzes the reduction of nitrate via nitrite to ammonia for the anabolic incorporation of nitrogen into bio-molecules [4]. Nitrate reduction can be performed with different purposes (a) nitrate assimilation: the utilization of nitrate as a nitrogen source for growth, (b) nitrate respiration: the generation of metabolic energy by using nitrate as a terminal electron acceptor (c) nitrate dissimilation: the dissipation of excess reducing power for redox balancing [5]. Thus, the importance of nitrate reductase in nitrogen fixation is known. The interaction of naturally occurring flavonoids to the enzyme is of significance in plant biology. Therefore, it is of interest to document the molecular docking based interaction analysis of nitrate reductase enzyme with known natural flavonoids.

## Methodology

### Sequence data

Protein sequence (226 residues) of UniProtKB - P0AF33 nitrate reductase was retrieved from Uniprot [6].

### Template search

A sequence similarity search was performed using the Protein BLAST [7] tool to identify the structural template from Protein Data Bank (PDB) for homology modeling [8]. The entry with PDB ID: 1Q16 having an identity of 72% with UniProtKB - P0AF33 was selected as a template for homology modeling.

### Sequence alignment

**Figure 1:**
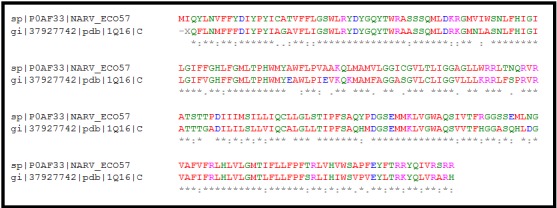
Sequence alignment of UniProtKB nitrate reductase with the known template structure (PDB ID: 1Q16) having 72% of identity and 84% similarity.

The online ClustalW tool [9] was used for sequence alignment. (Figure 1) shows the sequence alignment of UniProtKB - P0AF33 and template.

### Homology modeling

A homology model for UniProtKB - P0AF33 was subsequently generated using MODELLER version 9.16 [10]. The generated model was further checked for structure stereo-chemistry including Ramachandran plot and Psi/Phi angles using PROCHECK [11]

### Ligand structures

**Figure 2:**
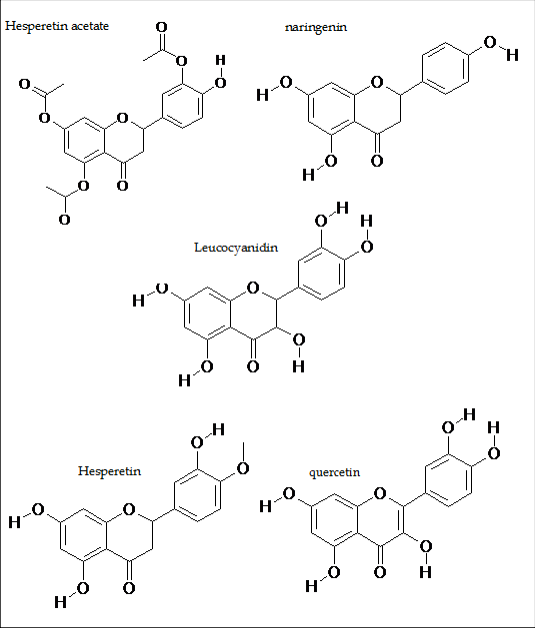
2D structures of natural flavonoids used for docking is shown

The structures of Musa paradiasica (common name: banana) extracted flavonoids Hesperetin (IUPAC Name: 5,7-Dihydroxy-2- (3-hydroxy-4-methoxy-phenyl)-chroman-4-one), Naringenin (IUPAC Name: 5,7-Dihydroxy-2-(4-hydroxy-phenyl)-chroman-4- one), Leucocyanidin (IUPAC Name: 2-(3,4-Dihydroxy-phenyl)- 3,5,7-trihydroxy-chroman-4-one), Quercetin (IUPAC Name: 2-(3,4- Dihydroxy-phenyl)-3,5,7-trihydroxy-chromen-4-one), Hesperetin triacetate (IUPAC Name: Acetic acid 5-[7-acetoxy-5-(1-hydroxyethoxy)- 4-oxo-chroman-2-yl]-2-hydroxy-phenyl ester) were shown (Figure 2). The ligand structures were sketched in SYBYL version 6.7 [12] and subsequently energy minimized. The structures were then saved in .mol2 format for further analysis.

### Molecular docking

Molecular docking studies were performed using the Autodock (version 4.2) software tool [13]. The structures were optimized by adding hydrogens using kollaman charges [14]. The model were prepared by optimizing torsion angles and saved in PDBQT format. Potential binding site for the nitrate reductase protein was identified using 3Dligand site [15]. A grid was generated to identify xyz coordinates (X=-149.455, Y=-6.672 and Z= -16.321) around the binding site of the enzyme. Lamarckian genetic algorithm (LGA) was selected for freezing, docking with default parameters in Autodock.

### Accessible surface area (ASA) versus residue number plot

ASA plot of nitrate reductase was completed using ASA-View, a database and tools for the solvent accessibility representation in proteins [17]. A characteristic 2D spiral plot of solvent accessibility provides a convenient graphical view of residues in terms of their exposed surface areas (Figure 6).

### Electrostatic distribution of the modeled surface

The electrostatic potential distribution of the nitrate reductase enzyme model was analyzed using UCSF Chimera (a highly extensible tool for the analysis of molecular structure) [16]. Electrostatic surface mapping of nitrate reductase was completed for distribution and charge related properties of the enzyme model. The surface of nitrate reductase was color coded as per the Coulomb’s law (Figure 7).

## Results and Discussion

**Figure 3:**
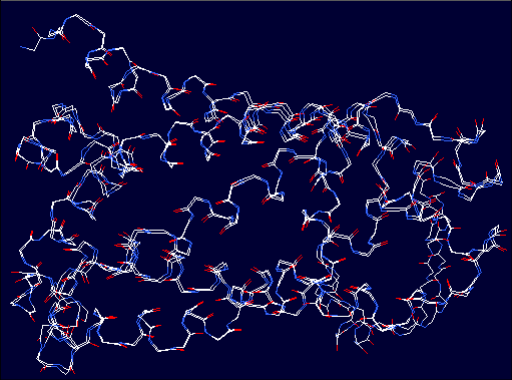
Superposed backbone traces for structures of the UniProtKB model and template (PDB ID: 1Q16). The structures were superimposed using SWISS PDB viewer (spdbv).

**Figure 4:**
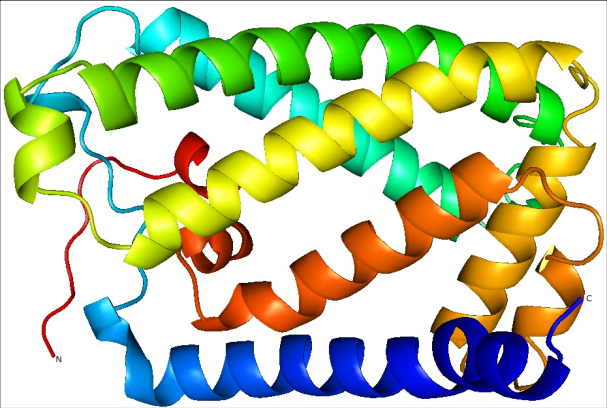
Cartoon representation of UniProtKB - P0AF33 nitrate reductase model with marked C terminal and N terminals.

A molecular model (Figure 4) of UniProtKB nitrate reductase enzyme (UniProtKB - P0AF33) was constructed using homology (Figure 3) modeling techniques (with crystal structure of nitrate reductase A, NarGHI, from Escherichia coli (PDB entry: 1Q16) as template structure (Figure 1) in MODELER version 9.16 and validated as described in the methodology section. The solvent accessible surface area (Figure 6) and the surface electrostatic distribution (Figure 7) of the enzyme model are presented. This information provides valuable insights to the physical and chemical features of the enzyme model towards its functional inference.

**Figure 5:**
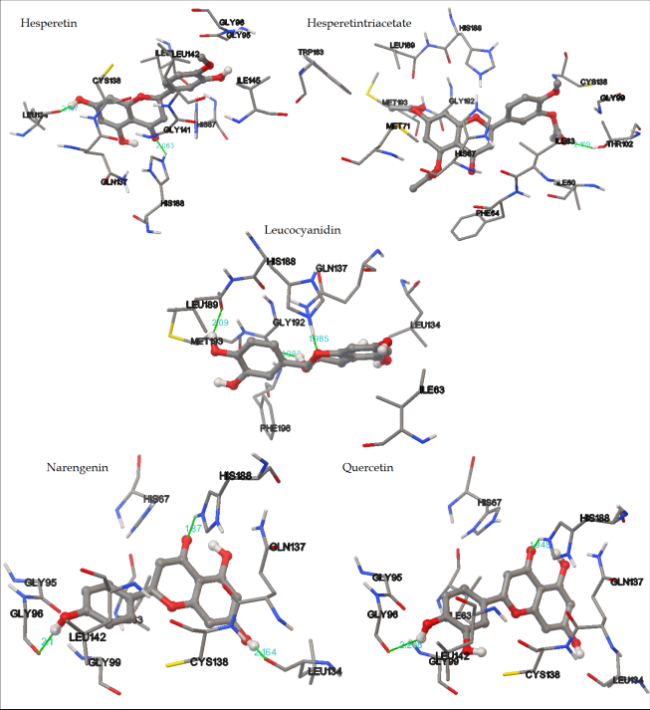
Docking interactions of UniProtKB - P0AF33 nitrate reductase model with known flavonoids such as hesperetin, hesperetintriacetate, leucocyanidin, narengenin and quercetin.

**Figure 6:**
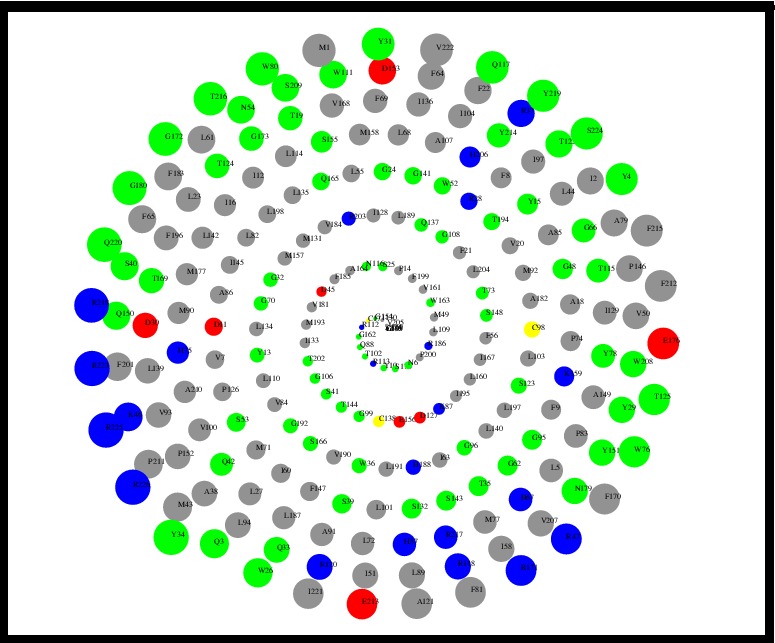
ASA vs residue number plot using ASA-View for nitrate reductase. The colors are coded as Blue for Positive charged residues (R, K, H), Red for Negative charged residue (D, E), Green for Polar uncharged residues (G, N, Y, Q, S, T, W), Yellow for Cystein and Gray for Hydrophobic residues (all others) for model.

**Figure 7:**
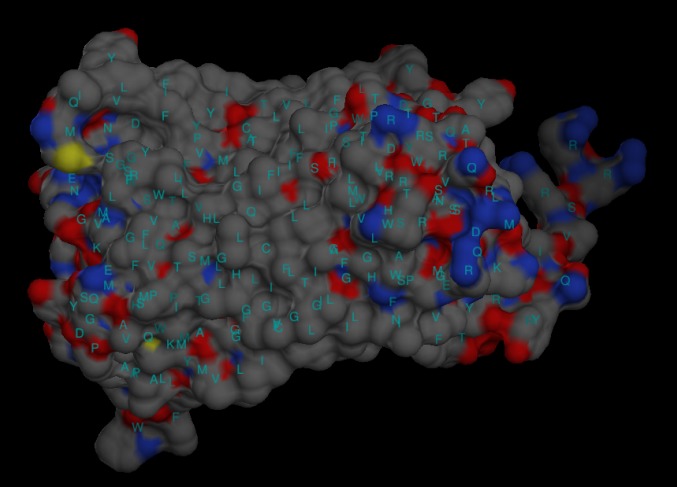
Electrostatic surface distribution of the modeled surface produced using UCSF Chimera. The surface was color coded as per the standard protocol of UCSF Chimera. Each amino acid was marked with standard code (blue for positive potential, white for neutral potential and red for negative potential).

The model was further used for the structural docking of five banana derived natural flavonoids (Figure 2). The molecular interactions of the five flavonoids with the nitrate reductase model are shown in (Figure 5). The characteristics binding (binding energy (ΔG) value, H bonds and docking score) of flavonoids to the reductase enzyme is given in Table 1 (see page 429). Among the five M. paradiasica derived secondary metabolites of hesperitin triacetate, naringenin, quercetin and hesperitin showed binding energy (ΔG) values of -8.36, -7.53, -7.32 and -6.72 kcal per mole, respectively. The compound leucocyanidin shows the least binding energy of -5.76 with three hydrogen-bonding interactions with Q137, G192 and L189.

Data shows the binding of hesperitin triacetate with the nitrate reductase protein model with notable features. Hesperitin triacetate shows a high binding energy of -8.36 kcal per mol and interacting with the residue T102 at a distance of 2.169 Å. A hydrogen bond was seen between hydrogen of T102 and the oxygen of hesperitin triacetate. Data shows that naringenin interacts with three amino acid residues H188, L134 and G96 with a docking score of -7.53 kcal per mol. It is also observed that quercetin shows a binding energy of -7.32 kcal per mol while interacting with H188 and G96.

## Conclusion

The binding characteristics of natural flavonoids such as hesperetin, naringenin, leucocyanidin, quercetin and hesperetin triacetate with the UniProtKB - P0AF33 structural model of nitrate reductase are documented in this study. The exercise shows that hesperitin triacetate having best binding features with the nitrate reductase protein model. This provides valuable insights towards the binding of natural flavonoids with the nitrate reductase enzyme and its importance in plant biology.

## Figures and Tables

**Table 1:** Hydrogen bond interactions of flavonoids with the nitrate reductase model

Ligand	Interacting amino acids	Grid X-Y-Z coordinates	Binding energy ΔG (Kcal/Mol)	Dissociation constant (kl) (μM)
Hesperetin acetate	Thr102	-149.455,-6.672-16.321	-8.36	749.04
Naringenin	His188,Leu135,Gly96	-149.455,-6.672-16.321	-7.53	3.02
Leucocyanidin	Gln137,Leu189,Gly192	-149.455,-6.672-16.321	-5.76	60.37
Quercetin	His188,Gly96	-149.455,-6.672-16.321	-7.32	4.34
Hesperetin	His188,Leu134	-149.455,-6.672-16.321	-6.72	11.95
